# Distinct roles of HMOX1 on tumor epithelium and macrophage for regulation of immune microenvironment in ovarian cancer

**DOI:** 10.1097/JS9.0000000000002829

**Published:** 2025-07-02

**Authors:** Yi Liu, Li-Jun Jiang, Hong-Fang Liu, Li Chen, Lei Guo, Jun Ge, Xin-Yi Zhang, Jing Li, Wei Gong

**Affiliations:** a Hubei Provincial Key Laboratory for Chinese Medicine Resources and Chinese Medicine Chemistry, School of Pharmacy, Hubei University of Chinese Medicine, Wuhan, China; b Hubei Shizhen Laboratory, Wuhan, China; c Key Laboratory of Chinese Medicinal Resource and Chinese Herbal Compound of the Ministry of Education, Wuhan, China; d Department of Hematology, Tongji Hospital, Tongji Medical College, Huazhong University of Science and Technology, Wuhan, Hubei, China; e Department of Oncology, Xiangyang Central Hospital, Hubei University of Arts and Science, Xiangyang, China

**Keywords:** ferroptosis, HMOX1, macrophage, ovarian cancer, PD-1 inhibitor

## Abstract

**Background:**

Ferroptosis has been implicated in the regulation of the tumor immune environment; however, its precise effect on immune checkpoint inhibitors remains contradictory.

**Objective:**

To elucidate the “double-edged sword effect” of a key ferroptosis-related factor in regulating the immune microenvironment.

**Methods:**

This study utilized single-cell RNA sequencing (scRNA-seq) analysis to characterize the tumor microenvironment in ovarian cancer samples from immunotherapy cohorts. Following quality control and variable gene screening, data from The Cancer Genome Atlas (TCGA), Genotype-Tissue Expression (GTEx), GENE EXPRESSION OMNIBUS (GEO), bulk, and spatial transcriptome databases were analyzed. The AddModuleScore_UCell function was employed for gene set scoring by evaluating the expression patterns of specific gene features in single-cell datasets, which were found to correlate with interactions between tumor cells and stromal cells, recognized as key contributors in the immunosuppressive milieu. Immunohistochemistry, western blot, and multiplex immunohistochemistry (mIHC) analyses were employed to explore the HMOX1/TGF-β1/PI3K/AKT/NF-κB(p65) signaling pathways. *In vitro* findings were further validated in a mouse model. The correlation between risk factors and progression-free survival (PFS) was analyzed using Cox regression and Kaplan–Meier methods.

**Results:**

We demonstrated decreased expression of the ferroptosis-activating gene *HMOX1* in ovarian cancer epithelial cells, while being upregulated in macrophages. Ovarian cancer (OV) epithelial cells with *HMOX1* inhibition could secrete TGF-β1 to activate three macrophage subtypes: SPP1^+^, FOLR2^+^, and C1QC^+^ via the PI3K/AKT/NF-κB (p65) pathway. The up-regulation of *HMOX1* in macrophages also activated these three macrophage subtypes via the NF-κB pathway. Both pathways simultaneously inhibited Cytotoxic T Lymphocyte (CTL) activation and contributed to the immunosuppressive microenvironment of ovarian cancer, as demonstrated in both *in vitro* and *in vivo* models. Targeting HMOX1 alone, whether through activation or inhibition, was only effective in modulating a single pathway while simultaneously inducing negative feedback on the opposing pathway, demonstrating the “double-edged sword effect” of HMOX1 in regulating the immune microenvironment.

**Conclusion:**

Overall, we proposed and validated two strategies targeting HMOX1 to improve the efficacy of PD-1 inhibitors, and confirmed that HMOX1, TGF-β1, SPP1, FOLR2, and C1QC could be used to construct models predicting the efficacy of immune checkpoint inhibitors.

## Introduction

Ovarian cancer, the most lethal gynecological malignancy, ranks as the fifth leading cause of death among women. Most cases are diagnosed at an advanced stage, often leading to relapse and drug resistance^[1,2]^. However, the 5-year survival rate remains below 40%^[3]^. In recent years, immune checkpoint therapy has emerged as one of the most promising approaches for cancer treatment^[4]^. This approach harnesses immune checkpoint inhibitors to activate tumor-infiltrating lymphocytes (TILs) and facilitate the eradication of tumor cells^[5]^. However, the effectiveness of this therapy varies depending on the infiltration status of TILs within the tumor microenvironment. Tumors can be histologically classified as either “hot tumors” or “cold tumors”^[6]^. Immune checkpoint inhibitors have demonstrated success in activating TILs in various “hot” tumors, such as melanoma, non-small cell lung cancer, renal cell cancer, bladder cancer, and Hodgkin’s lymphoma^[7]^, given that a higher abundance of TILs has been associated with improved clinical prognosis^[8]^. Despite many ovarian cancer patients exhibiting a higher number of TILs and elevated PD-L1 expression, the efficacy of immune checkpoint inhibitors remains poor^[9]^, which may be attributed to the presence of an immunosuppressive network, which is also a key factor in classifying ovarian cancer as a “cold” tumor^[10]^. Therefore, it is imperative to investigate the key molecular targets responsible for inducing resistance to immune checkpoint inhibitors and elucidate their underlying regulatory mechanisms. Addressing this urgent issue holds significant potential for improving the clinical treatment outcomes and survival rates of ovarian cancer patients.

Recent research has demonstrated that ferroptosis in tumors induces an immunosuppressive environment by inhibiting T cell activity, thereby contributing to resistance to immune checkpoint inhibitors^[11]^. Most studies have shown that inhibition of ferroptosis in tumor cells induces an immunosuppressive environment, and ferroptosis activators could improve the efficacy of immune checkpoints^[12–14]^. On the other hand, some studies on immune cells have shown that during tumor development, the immune system’s ferroptosis is activated, and ferroptosis inhibitors can enhance the efficacy of immune checkpoints^[15,16]^. These investigations suggest that ferroptosis may exert a “double-edged sword effect” on the tumorigenesis process and the efficacy of immune checkpoint inhibitors across different tumor tissue cells. Therefore, the integrated analysis of bulk, single-cell and spatial transcriptomics may provide a more comprehensive understanding of ferroptosis gene expression and its intricate relationship with the immune microenvironment in various cell types, offering valuable insights for the development and clinical use of immune checkpoint inhibitors.

In the present study, transcriptome analysis revealed that HMOX1 was inhibited in PD-1 inhibitor-resistant ovarian cancer cells. Besides, HMOX1 expression was lower in TCGA-OV than in GETx samples. Interestingly, analysis of dataset GSE40595 revealed higher HMOX1 expression in the ovarian cancer stroma than in the normal ovarian stroma, but lower expression in the ovarian cancer epithelial compartment than in the normal ovarian epithelial compartment. Spatial transcriptome analysis showed that HMOX1 expression was lower in ovarian cancer tissues than in borderline tissues, and lower in borderline tissues than in normal tissues. Single-cell analysis showed that HMOX1 was mainly expressed in macrophages. Pseudo-time series analysis revealed that ovarian cancer epithelial cells with low HMOX1 expression and macrophages with high HMOX1 expression became the dominant cells as the tumor progressed. By using protein microarray, transcriptome sequencing, and single cell analysis, we demonstrated that decreased HMOX1 expression in ovarian cancer epithelium and increased HMOX1 expression in macrophages can activate three macrophage subtypes: SPP1^+^, FOLR2^+^, and C1QC^+^ through the NF-κB pathway, thereby inhibiting CD8T^+^ cell activation. Finally, HMOX1, TGF-β1, SPP1, FOLR2, and C1QC were used to construct a prediction model for the efficacy of immune checkpoint inhibitors in ovarian cancer. Moreover, two treatment strategies combined with PD-1 inhibitors were provided to overcome the “double-edged sword effect” of HMOX1. Overall, this study confirmed that ferroptosis gene expression in different cells of ovarian cancer exhibits different regulatory directions in terms of resistance to immune checkpoint inhibitors, and this phenomenon may be prevalent in clinical practice yet frequently overlooked. Therefore, the findings of this study offer novel insights and directions for clinical research and the translation of research outcomes. The entire study followed the TITAN guideline, and AI was used in the research and manuscript development^[17]^.

**HIGHLIGHTS**

- Ovarian cancer epithelial cells with HMOX1 inhibition secrete TGF-β1 to activate three macrophage subtypes of SPP1, FOLR2, and C1QC.
- The up-regulation of HMOX1 on macrophages activates three macrophage subtypes of SPP1, FOLR2, and C1QC.
- HMOX1 has a double-edged sword effect in different cells of ovarian cancer and are targets therapy in OV patients with PD-1 inhibitor.
- The HMOX1/TGF-β1/PI3K/AKT/NF-ΚB/SPP1/FOLR2/C1QC axis establishes OV immunosuppressive environment conducive to PD-1 inhibitor resistance.

## Materials and methods

### Data acquisition and processing

Data for this study were acquired and processed from the following publicly available datasets: GSE40595 and TCGA-OV for bulk RNA sequencing, GSE154600 for single-cell RNA sequencing (scRNA-seq), and GSE203612 for spatial transcriptomics.

### Transcriptome sequencing

Transcriptome sequencing was done for ID8 versus ID8R.

Transcriptome sequencing was done for RAW264.7 cells (from uncontact-cultured with shHMOX1 ID8 cells) versus RAW264.7 cells (from uncontact-cultured with HMOX1-OE ID8 cells).

### Tumor immune dysfunction and exclusion (TIDE)

A framework has been developed for assessing tumor immune evasion capabilities, achieved through the analysis of gene expression data derived from tumor specimens. The comprehensive dataset encompasses approximately 30 000 gene expression profiles, spanning across 166 distinct cancer types^[18]^.

### Kaplan–Meier survival curve analysis

Kaplan–Meier plotter (http://kmplot.com/analysis/) is an open-access online tool for prognostic meta-analysis, capable of analyzing the expression of 54k genes on survival across 21 cancer types^[19]^. In this study, Kaplan–Meier plots were generated to examine the relationship between expression of target genes and clinic survival rates in OV based on hazard ratios (HRs) of 95% confidence intervals (CIs) and the log-rank *P* value. The “Immunotherapy” module was used to analyze the correlation between anti-PD-1 resistance and target genes in many cancer types.

### TISCH2

Tumor Immune Single-cell Hub 2 (TISCH2) is a scRNA-seq database focusing on the tumor microenvironment (TME). TISCH2 provides detailed cell-type annotation at the single-cell level, facilitating comprehensive exploration of the TME across different cancer types^[20]^.

### Gene set enrichment analysis (GSEA)

GSEA is a computational method that identifies statistically significant, concordant differences in a predefined set of genes between two biological states^[21]^. In the present study, GSEA was conducted on cancer stroma from GSE40595, comparing the HMOX1-high stroma group with the HMOX1-low stroma group.

### Reagents and cells

The antibodies for NOX1(ab131088), HMOX1(ab137749), NF-κB(p65) (ab32536), NF-κB(p-p65) (ab86299), AKT (ab8805), p-AKT (ab38449), ERK (ab184699), p-ERK (ab201015) were purchased from Abcam. P38 (GB114685-100) and p-P38 (GB113380-100) were acquired from Servicebio. Recombinant human TGF beta 1 protein (ab50036), IMD-0354 (ab144823), LY294002 (ab120243), U0126 (ab120241), SB203580 (ab120162), PDTC (ab141406), Mouse SPP1 ELISA Kit (ab100734), Mouse TGF beta 1 ELISA Kit (ab119557), Mouse TNF alpha ELISA Kit (ab208348), Mouse TNF beta ELISA Kit (ab315497), Mouse IL-10 ELISA Kit (ab255729), and the Mouse IL-12 ELISA Kit (ab100699) were obtained from Abcam. The Mouse FOLR2 ELISA Kit (E7165m) was purchased from SHRBIO, and the Mouse C1QC ELISA Kit (QT-EM0450) was from FineTest. TGF-β1 inhibitor (HY-P0118A), anti-PD-1 antibody (HY-P99144), HMOX1 activator (Carnosol, HY-N0643), and HMOX1 inhibitor (ZnPP, HY-101193) were obtained from MCE. The antibodies for horseradish peroxidase conjugated anti-mouse IgG and anti-rabbit IgG were bought from Epitomics. A Human Cytokine Antibody Array (AAH-CYT-5) was acquired from RayBiotech. All overexpression plasmids and lentivirus were purchased from Sangon Biotech. All cells were grown in DMEM medium supplemented with 10% fetal bovine serum (FBS), 100 U/ml penicillin, 100 μg/ml streptomycin and 25 μg/ml amphotericin B. Cells were cultured at 37 °C in a humidified incubator with 5% CO_2_ and 95% air.

### Western blot analysis

Cells were collected and lysed in RIPA buffer (50 mM Tris–Hcl, 1 mM EDTA, 150 mM NaCl, 1% NP-40) containing 1 mM PMSF and a protease inhibitor cocktail. After 30 min on ice, the mixtures were centrifuged at 12 000 rpm for 15 min, and the supernatant was harvested. The concentration of each sample was determined by using the Bio-Rad protein assay reagent. 50 μg of total protein from each sample was separated by SDS–PAGE gel, and then was electrophoretically transferred to a PVDF membrane. After the transfer, the membranes were blocked with TBS containing 5% non-fat dry milk at room temperature for 1 h. Then the membranes were incubated with the primary antibody at 4°C overnight, followed by incubation with the HRP-linked secondary antibody. Finally, the immune bands were visualized via fluorography using an enhanced ECL system.

### Enzyme-linked immunosorbent assay

ELISA kits were utilized in accordance with the manufacturers’ protocol.

#### *In vivo* experiments

This study was performed with the approval of the Committee on the Ethics of Animal Experiments of the corresponding author’s hospital. All animal experiments were carried out in accordance with the Guide for the Care and Use of Laboratory Animals of the corresponding author’s hospital. All animal experiments were conducted in accordance with the ARRIVE guidelines^[22]^.

For the *in vivo* anti-PD1 resistant cell, 5 × 10^5^ ID8 cells were inoculated into the C57BL/6 mice. αPD1 was administered to the mice 5 times at 3-day intervals. Tumors were harvested and dissociated for culturing. The treatment cycle was repeated 2 more times to obtain ID8R cells. Next, 5 × 10^5^ ID8 cells or ID8R cells were intraperitoneally injected into mice. After inoculation with the tumor, mice were treated with isotype control or αPD1 antibody (200 µg i.p. injection every 3 days) for a total of 5 doses. Each experimental group comprised 8 mice. Mice were then euthanized, and tumors were dissected and weighed.

5 × 10^5^ ID8 cells were subcutaneously injected into C57BL/6 mice. After inoculation with the tumor, mice were randomly assigned to one of the following treatment groups: isotype control, αPD1 antibody (200 µg i.p. injection every 3 days), Carnosol (200 mg/kg i.p. injection every 3 days), αPD1 antibody (200 µg i.p. injection every 3 days) + Carnosol (200 mg/kg i.p. injection every 3 days). Each treatment regimen consisted of a total of 5 doses. Each experimental group comprised 8 mice. Mice were then euthanized, and tumors were dissected and weighed.

5 × 10^5^ ID8 cells were subcutaneously injected into C57BL/6 mice. After tumor inoculation, mice randomly assigned to receive one of the following treatments for a total of 5 doses (n =8 mice/group): isotype control, αPD1 antibody (200 µg i.p. injection every 3 days), TGF-β1 inhibitor (10 mg/kg i.p. injection every 3 days), αPD1 antibody (200 µg i.p. injection every 3 days) plus TGF-β1 inhibitor (10 mg/kg i.p. injection every 3 days), LY294002 (10 mg/kg i.p. injection every 3 days) or αPD1 antibody (200 µg i.p. injection every 3 days) plus LY294002 (10 mg/kg i.p. injection every 3 days). Mice were subsequently sacrificed, and tumors were dissected and weighed.

5 × 10^5^ ID8 plus RAW264.7 cells or ID8 plus HMOX1-OE RAW264.7 cells were subcutaneously injected into C57BL/6 mice. After tumor inoculation, mice were treated with either the isotype control or αPD1 antibody (200 µg i.p. injection every 3 days) for a total of 5 doses (n =8 mice/group). Mice were subsequently sacrificed, and tumors were dissected and weighed.

5 × 10^5^ ID8 cells were subcutaneously injected into C57BL/6 mice. After tumor inoculation, mice were randomly assigned to one of the following treatment groups for a total of 5 doses (n =8 mice/group): IgG, PD-1 blocking antibody (200 µg i.p. injection every 3 days), TGF-β1 blocking antibody (10 mg/kg i.p. injection every 3 days), ZnPP (200 mg/kg i.p. injection every 3 days), TGF-β1 blocking antibody + PD-1 blocking antibody, ZnPP + PD-1 blocking antibody or ZnPP + TGF-β1 blocking antibody + PD-1 blocking antibody. Mice were then sacrificed, and tumors were dissected and weighed.

5 × 10^5^ ID8 cells were subcutaneously injected into C57BL/6 mice. After tumor inoculation, mice were randomly assigned to one of the following treatment groups for a total of 5 doses (n =8 mice/group): IgG, PD-1 blocking antibody (200 µg i.p. injection every 3 days), IMD0354 (10 mg/kg i.p. injection every 3 days), carnosol (200 mg/kg i.p. injection every 3 days), IMD0354 + PD-1 blocking antibody, carnosol + PD-1 blocking antibody or carnosol + IMD0354 blocking antibody + PD-1 blocking antibody. Mice were subsequently sacrificed, and tumors were dissected and weighed.

### Single-cell transcriptome analysis

The Seurat package (V.5.1.0) was employed for downstream analysis. Cells with fewer than 500 UMIs, greater than 25% mitochondria genes, less than 3% ribosome genes, and greater than 1% HB genes were excluded from the analysis. Principal component analysis (PCA) was conducted using these variable genes. Harmony was applied to remove batch effects between samples. Nearest neighbors for graph-based clustering were determined using the *FindNeighbors* function. The resulting clusters were obtained using the *FindCluster* function, followed by visualization via t-SNE. Furthermore, gene signatures specific to various cell types were scored across clusters. These gene signatures included markers for B cells (MS4A1, CD19, CD79A, IGHG1, MZB1 and SDC1), endothelial cells (CDH5, PECAM1 and VWF), Epithelial (EPCAM, KRT19 and CLDN4), Fibro (PECAM1, CLO1A2, VWF, LUM, FGF7 and MME), Macrophage (CSF1R, CSF3R and CD68), Mast (CPA3, CST3, KIT, TPSAB1 and TPSB2), T (CD3D, CD3E, CD8A, CD4 and CD2), NK (KLRD1, GNLY, KLRF1, AREG, XCL2 and HSPA6) and Proliferative (MKI67, STMN1 and PCNA). Differential gene expression analysis between clusters was performed using the *FindAllMarkers* function, with the following parameters: “min.pct = 0.25,” “thresh.use = 0.25,” and “only.pos = TRUE.”

Macrophage subsets were further classified based on distinct gene signatures, including markers for SPP1^+^ cells (SPP1, MARCO, and FBP1), FOLR2^+^ cells (FOLR2, LYVE1, and SELENOP), MT^+^ cells (MT1H, MT1G, and MT1X), MKI67^+^ cells (MKI67, TOP2A, and PCLAF), IL1B^+^ cells (IL1B, IL1A, NLRP3), and C1QC^+^_MRC1^−^ cells (C1QC, MRC1, and VCAN).

### Spatial transcriptomics analysis

Seurat version 5.1.0 was used for the analysis of the gene-spot matrix, which was derived after processing spatial transcriptomics (ST) data. The spots were subjected to filtration, ensuring a minimum detection threshold of 200 genes. Concurrently, genes that were expressed in fewer than three spots were eliminated. Normalization across the spots was executed employing the LogVMR function. Subsequently, dimensionality reduction and clustering were conducted using independent component analysis (ICA), focusing on the initial 30 principal components (PCs). To accentuate the spatial expression of features, the spots were augmented through the application of the *spatialEnhance* function from the BayesSpace package, version 1.6.0. Besides, the *enhanceFeatures* function was used to intensify the expression features. The signature score, derived from the single-cell RNA sequencing (scRNA-seq) dataset, was integrated into the “metadata” of the ST dataset using the *AddModulScore* function in Seurat, with default settings. Spatial feature expression plots were crafted using the *SpatialFeaturePlot* function within the Seurat package. The SpaGene methodology was implemented to identify ten distinct spatial gene expression patterns per sample, and the similarity of these patterns was assessed using the Jaccard index. The findings were visualized using the ComplexHeatmap package, version 2.12.1.

### Trajectory analysis

We employed the Monocle (v2.28.0) algorithm to construct the single-cell pseudotime trajectory of epithelial cell subtypes^[15]^. The trajectory branch exhibiting high differentiation scores and low dedifferentiation scores was selected as the root state.

### CytoTRACE

We employed the CytoTRACE (v1.0.0) computational framework to predict the developmental potential of cells by analyzing the expression levels of genes^[23]^. This method leverages the inverse relationship between the number of expressed genes per cell and transcriptional diversity to infer differentiation states from scRNA-seq data.

### Immunofluorescence

4-μm-thick sections of human ovarian/OV samples were deparaffinized and rehydrated through a graded alcohol series. Antigen epitope retrieval solution (citrate buffer, pH = 6) was preheated, and 3% H_2_O_2_ was incubated for 20 minutes to quench endogenous peroxidase. Next, the slides were pre-incubated with 10% normal goat serum for 20 minutes and then incubated overnight with primary antibodies from three panels: EpCAM, CD11b, SPP1, HMOX1, C1QC, FOLR2, and TGF-β1, respectively. Then, the corresponding HRP conjugated secondary antibodies and fluorescent dyes were applied to each antibody in the following order: Opal-540, Opal-650, Opal-480, Opal-570, Opal-620, Opal-690, Opal-520, respectively. The nuclei were counterstained with DAPI.

Multiplex immunofluorescence (mIFC) slides were imaged using the Vectra Polaris (v1.0; Akoya Bioscience) platform. Single-stained tissue sections for each reagent were scanned to generate spectral libraries to unmix the multispectral images by using Inform Advanced Image Analysis software (Inform v2.0; Akoya Bioscience). Tissue segmentation, cell segmentation, and phenotyping were performed with Inform Advanced

### Statistical analysis

The data were presented as means ± standard deviation (SD). All experiments were done at least three times and evaluated by one-way ANOVA. Statistical analysis was performed using SPSS v22.0. A *P* value <0.05 was considered statistically significant.

## Results

### Inhibition of HMOX1 involved in the resistance of PD-1 inhibitors in ovarian cancer

**Figure 1.**
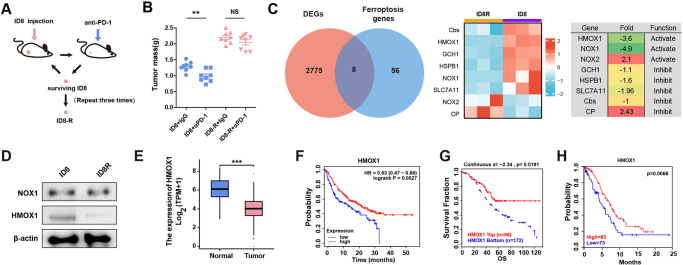
**Inhibition of HMOX1 involved in PD-1 inhibitor resistance in ovarian cancer. (A)** Schematic illustrating the establishment of anti-PD-1 resistant cells. **(B)** ID8 and ID8R tumor-bearing mice treated with anti-PD-1(αPD-1) or IgG. N = 8 for each group. **(C)** Ferroptosis gene sets obtained from the Molecular Signatures Database. DEGs were intersected with ferroptosis genes. **(D)** Western blot analysis of the expression of NOX1, HMOX1, and β-actin in ID8 and ID8-R cells. **(E)** Comparison of HMOX1 expression between TCGA-OV and GETx-ovary. **(F)** Overall survival of cancers with high and low HMOX1 mRNA expression that received anti-PD-1 inhibitor therapy was analyzed by KM-plot. **(G)** Overall survival of patients with ovarian cancer with high and low HMOX1 mRNA expression who received anti-PD-1 antibody therapy (TIDE). **(H)** Overall survival of 156 patients with ovarian cancer with high and low SPP1 mRNA expression who received anti-PD-1 antibody therapy. **P < 0.01.

To investigate mechanisms of PD-1 inhibitor resistance in ovarian cancer, we established a drug-resistant mouse tumor model (Fig. 1A). We subcutaneously injected either ID8 or ID8R into the hind limbs of C57BL/6 mice, which were subsequently treated with anti-PD-1 antibodies. As depicted in Figure 1B, anti-PD-1 antibody treatment effectively inhibited tumor growth in the ID8 model compared to IgG control. However, no significant difference was observed between IgG and anti-PD-1 antibody treatment in the ID8R model, indicating ID8R as a PD-1 inhibitor-resistant cell line. To identify key ferroptosis genes associated with resistance to PD-1 inhibitors, ID8 and ID8R cells underwent transcriptome sequencing analysis. As shown in Figure 1C, eight ferroptosis-related genes exhibited more than a two-fold upregulation or downregulation in ID8R cells compared to ID8 cells. To confirm the status of the ferroptosis pathway in ovarian cancer and PD-1 inhibitor resistance, we examined ROS production in ID8 and ID8R cells and compared the expression of several key ferroptosis genes between TCGA and GTEx datasets. Higher levels of GPX4 and SLCA711 were observed in ovarian tumor tissue compared to normal ovarian tissue based on TCGA and GTEx datasets (Supplemental Digital Content Figure S1A, available at: http://links.lww.com/JS9/E551). ROS production in ID8R cells was significantly lower than in ID8 cells (Supplemental Digital Content Figure S1B, available at: http://links.lww.com/JS9/E551), which indicated that the ferroptosis pathway was mainly inhibited in ovarian cancer, contributing to PD-1 inhibitor resistance. HMOX1 and NOX1, key ferroptosis activation genes, were verified by Western blot analysis. Only the protein expression level of HMOX1 was reduced in ID8R cells (Fig. 1D). Comparative analysis between TCGA and GTEx datasets revealed lower mRNA levels of HMOX1 in ovarian tumor tissue compared to normal ovarian tissue (Fig. 1E). Data from the KM-plotter database revealed that HMOX1 expression positively correlated with the efficacy of immune checkpoint inhibitors in multiple cancers (Fig. 1F). Furthermore, we analyzed the correlation between HMOX1 expression and PD-1 inhibitor resistance using the TIDE online database. We identified a positive correlation between HMOX1 expression and overall survival in ovarian cancer patients treated with PD-1, PD-L1, and CTLA-4 inhibitors (Fig. 1G). To validate this finding, we retrospectively analyzed ovarian cancer patients treated with PD-1 inhibitors between 2018 and 2022. Patient characteristics at baseline are summarized in Table 1. Decreased HMOX1 expression was significantly associated with elevated serum carbohydrate antigen 12-5 levels, presence of surgery, and more advanced tumor stages, indicating a correlation between HMOX1 and OV progression (Table 1). Furthermore, Kaplan–Meier survival analysis revealed a positive association between HMOX1 expression and patient progression-free survival (Fig. 1H).

**Table 1:** General characteristics of ovarian cancer patients

Characteristics	Total (n = 156)	Low HMOX1 (n = 72)	High HMOX1 (n = 84)	*P* value
Age, Mean, n(%)				0.748
≤58	78 (50.00)	37 (51.39)	41 (48.81)	
>58	78 (50.00)	35 (48.61)	43 (51.19)	
Primary location, n (%)				0.024
Left	47 (30.13)	18 (25.00)	29 (34.52)	
Right	54 (34.62)	33 (45.83)	21 (25.00)	
Unknown	55 (35.26)	21 (29.17)	34 (40.48)	
Histological type, n (%)				0.509
High and moderate	37 (23.72)	14 (19.44)	23 (27.38)	
Low	78 (50.00)	38 (52.78)	40 (47.62)	
Undifferentiation	41 (26.28)	20 (27.78)	21 (25.00)	
TNM stage, n (%)				<0.001
III stage	75 (48.08)	45 (62.50)	30 (35.71)	
IV stage	81 (51.92)	27 (37.50)	54 (64.29)	
Previous surgery, n (%)				0.510
No	122 (78.21)	58 (80.56)	64 (76.19)	
Yes	34 (21.79)	14 (19.44)	20 (23.81)	
Previous line of chemotherapy, n (%)				0.233
2rd line therapy	70 (44.87)	36 (50.00)	34 (40.48)	
Above 3rd line therapy	86 (55.13)	36 (50.00)	50 (59.52)	
Lymph node metastasic, n (%)				0.831
No	107 (68.59)	50 (69.44)	57 (67.86)	
Yes	49 (31.41)	22 (30.56)	27 (32.14)	
Expression of PD-L1, n (%)				0.621
≤5 cm	77 (49.36)	34 (47.22)	43 (51.19)	
˃5 cm	79 (50.64)	38 (52.78)	41 (48.81)	
Size of tumor, n (%)				0.136
≤5 cm	110 (70.51)	55 (76.39)	55 (65.48)	
>5 cm	46 (29.49)	17 (23.61)	29 (34.52)	
CEA, n (%)				0.404
≤5	103 (66.03)	50 (69.44)	53 (63.10)	
>5	53 (33.97)	22 (30.56)	31 (36.90)	
CA125, n (%)				0.324
≤30	102 (65.38)	50 (69.44)	52 (61.90)	
>30	54 (34.62)	22 (30.56)	32 (38.10)	

### Inhibition of HMOX1 in tumor epithelium reduces the ability of CTL to induce resistance to PD-1 inhibitors in ovarian cancer

**Figure 2.**
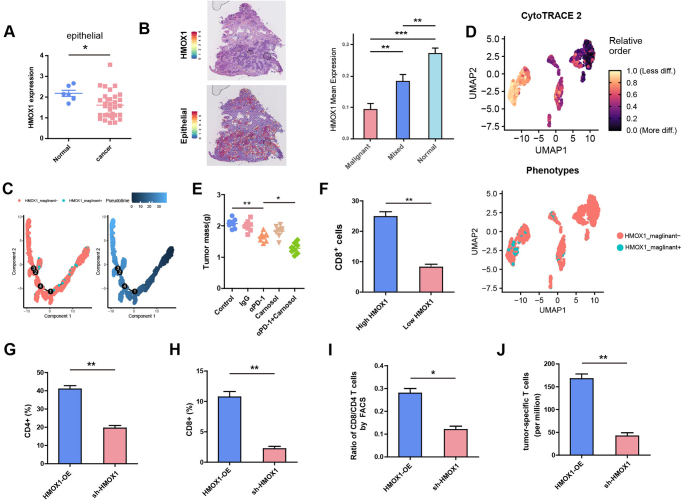
**Tumor epithelial HMOX1 inhibition attenuates cytotoxic T lymphocyte activity and contributes to PD-1 inhibitor resistance in ovarian cancer. (A)** The expression of HMOX1 in tumor epithelial cells compared with normal epithelial cells in GSE40595. **(B)** Tumor cell spots and HMOX1 spots in GSE203612 shown. HMOX1 mean expression of malignant, mixed and normal in GSE203612 was analyzed. **(C)** Monocle plots showing the differentiation trajectories of HMOX1_malignant^+^ and HMOX1_malignant^−^ cells. (D) Combined application of CytoTRACE to dissect HMOX1_malignant^+^ and HMOX1_malignant^−^ cells. (E) Tumor growth in ID8 tumor-bearing mice treated with saline, IgG, αPD-1, carnosol or αPD-1 + carnosol (n = 8 mice/group). Tumor growth was monitored by measuring the tumor volume for 2 weeks. (F) Cytotoxic CD8^+^ T cells were analyzed in the high HMOX1 expression group compared to the low HMOX1 expression group in 156 samples. (G-I) A tumor-bearing mouse model was established, including two groups: HMOX1-OE ID8 group, sh-HMOX1 ID8 group. (G) The percentage of CD4^+^ T cells was calculated in two groups. (H) The percentage of CD8^+^ T cells was calculated in two groups. (I) The ratio of CD8^+^/CD4^+^ T cells was calculated in two groups. (J) The number of tumor-specific T cells per million splenocytes in in two groups. *P < 0.05. **P < 0.01.

To explore the mechanism by which HMOX1 induces ovarian cancer resistance to PD-1 inhibitors, we first determined HMOX1 expression in different tumor cell types. Analysis of dataset GSE40595, which included four different clinical specimens (normal ovarian stromal, ovarian stromal, human ovarian surface epithelium, and tumor epithelial components), revealed lower HMOX1 expression in tumor epithelial cells than in normal epithelial cells (Fig. 2A). Spatial transcriptome analysis of dataset GSE203612 confirmed that HMOX1 was mainly upregulated in stromal cells surrounding tumor cells, with the highest expression found in normal tissues (Fig. 2B). These results suggested that the downregulation of HMOX1 in ovarian cancer compared with normal ovarian tissue is mainly due to the downregulation of HMOX1 in tumor epithelial cells compared with normal epithelial cells.

To confirm this result, we analyzed the scRNA-seq dataset GSE154600. Through rigorous quality control (Supplemental Digital Content Figure S2A, available at: http://links.lww.com/JS9/E551) and variable gene screening (Supplemental Digital Content Figure S2B, available at: http://links.lww.com/JS9/E551), we identified 9 cell categories: B cells, T cells, Endothelial cells, Epithelial cells, Fibroblasts, Macrophages, Mast cells, NK cells, and proliferative cells. Differential gene analysis was performed between TCGA-OV and GTEx-ovary under |log2 fold change| > 1 and *P* value <0.05 (Supplemental Digital Content Figure S2C, available at: http://links.lww.com/JS9/E551). Epithelial cells were subsequently subset from all cells for quality control according to Seurat V5 standard procedure (Supplemental Digital Content Figure S2D, available at: http://links.lww.com/JS9/E551). The AddModuleScore_UCell function enables gene set scoring by evaluating the expression patterns of specific gene features in single-cell data sets. The top 50 upregulated differential genes contributed to a “malignant score,” while the top 50 downregulated differential genes contributed to a “normal score,” thereby allowing epithelial cells to be classified as either malignant or non-malignant (Supplemental Digital Content Figure S2E, available at: http://links.lww.com/JS9/E551). For malignant epithelial cells, those expressing HMOX1 were designated HMOX1_malignant^+^, while those without HMOX1 were termed HMOX1_malignant^−^. To gain insights into the dynamic processes underlying epithelial subtypes at the single-cell level, we derived the pseudotime cell trajectory of HMOX1_malignant^+^ and HMOX1_malignant^−^ based on the Monocle 2 algorithm. HMOX1_maglinat^+^ was present mainly in the progenitor state, while HMOX1_malignant^−^ was present in the terminal state (Fig. 2C). To further determine the developmental trajectories of HMOX1 in epithelial subtypes, we applied CytoTRACE to delineate cellular hierarchies based on HMOX1_malignant^+^ and HMOX1_malignant-. The results showed a scarcity of HMOX1_malignant + cells in more differentiated stages, confirming that HMOX1_malignant^−^ plays a dominant role in the late stage of tumor development (Fig. 2D). Subsequently, a tumor-bearing mouse model was developed utilizing ID8 and treated with IgG, anti-PD-1(αPD-1), Carnosol, or αPD-1 + Carnosol. As illustrated in Figure 2E, activation of HMOX1 by Carnosol could enhance the effect of the PD-1 inhibitor.

Immune checkpoint inhibitors are used to treat tumors by blocking immunosuppressive signals, enhancing T cell activity, and restoring T cell immune killing function^[24]^. Besides, the deletion or inactivation of cytotoxic T lymphocytes within tumors has been linked to reduced overall survival^[25]^. Analysis of our clinical samples revealed a significant reduction in cytotoxic CD8^+^T cells in the low HMOX1 expression group compared to the high HMOX1 expression group (Fig. 2F). In a tumor-bearing mouse model established 14 days post-tumor inoculation, the sh-HMOX1 ID8 group exhibited markedly lower proportions of CD4^+^ and CD8^+^ T cells in all spleen cells, with a decreased CD4^+^T/CD8^+^T ratio compared to the ID8 combined with HMOX1-OE ID8 group (Fig. 2G-2I). Furthermore, the abundance of tumor-specific T cells per million spleen cells in the sh-HMOX1 ID8 group was notably diminished compared to the HMOX1-OE ID8 group (Fig. 2J). During tumor development, the gradual loss of HMOX1 in the epithelium of ovarian cancer tumors contributes to resistance to PD-1 inhibitors, suggesting that inhibition of HMOX1 in tumor epithelial cells may hinder the antitumor efficacy of PD-1 inhibitors by diminishing cytotoxic T cell activity.

### Inhibition of HMOX1 on tumor epithelium cell secreted TGF-β1 to active M2-type macrophages

**Figure 3.**
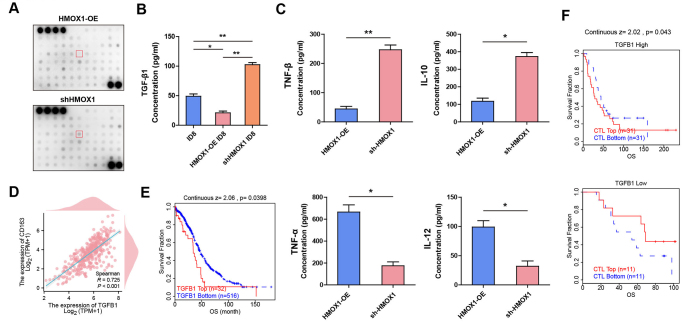
**Inhibition of HMOX1 in tumor epithelial cells promotes TGF-β1 secretion to activate M2-type macrophages. (A)** Cytokines were detected by AAH-CYT-5 between shHMOX1 cells and HMOX1-OE cells. **(B)** The concentrations of TGF-β1 in ID8, shHMOX1 cells or HMOX1-OE cells were detected by ELISA. **(C)** The concentration of TNF-α, IL-10, IL-12, and TNF-β in the supernatant of the shHMOX1 cell coculture with RAW264.7 or HMOX1-OE cell coculture with RAW264.7 was detected by ELISA. **(D)** Correlation analysis of mRNA levels of TGF-β1 with CD163 in TCGA-OV. **(E)** Overall survival of patients with ovarian cancer with high and low TGF-β1 gene copy numbers who received anti-PD-1 antibody therapy (TIDE). **(F)** Relationship between overall survival and CTL levels in patients with breast cancer with high and low SPP1 mRNA expression (TIDE). *P < 0.05. **P < 0.01.

To identify which cytokines are secreted by sh-HMOX1 ovarian cancer cells, we used cytokine protein chips to compare conditioned medium (CM) obtained from HMOX1-OE and sh-HMOX1 cells. As illustrated in Figure 3A, TGF-β1 expression was markedly higher in the CM from sh-HMOX1 cells compared to that from HMOX1-OE cells. Furthermore, TGF-β1 concentration in the supernatant of sh-HMOX1 ID8 cells was significantly elevated compared with that of ID8, while it was notably decreased in the supernatant of HMOX1-OE cells when compared to ID8 (Fig. 3B). Given that TGF-β1 is a key factor in the regulation of macrophage phenotypes in the tumor microenvironment^[26]^, IL-10, TNF-α, IL-12, and TNF-β were detected by ELISA to evaluate the relationship between HMOX1 in ovarian tumor epithelium cells and macrophage polarization. Notably, the concentration of IL-10 and TNF-β from the sh-HMOX1 group was significantly higher than that from the HMOX1-OE group, while the results for TNF-α and IL-12 displayed an inverse pattern (Fig. 3C). Furthermore, a strong positive correlation in mRNA expression between CD163 with TGF-β1 was observed in TCGA-OV (Fig. 3D), which suggested that HMOX1 inhibition in ovarian cancer epithelial resulted in TGF-β1 secretion, promoting M2 macrophage polarization.

TIDE database analysis also demonstrated a negative association between TGF-β1 levels and the activity of cytotoxic T lymphocytes or overall survival rates in ovarian cancer treatment by immune checkpoint inhibitors, as shown in Figures 3E and 3F. These data underscore that the potential of HMOX1/TGF-β1 in ovarian cancer epithelial cells to serve as an important signaling pathway for activating M2-type macrophages to influence the efficacy of immune checkpoint inhibitors.

### Role of TGF-β1 in activating SPP1^+^, FOLR2^+^, and C1QC^+^ subtype macrophages

**Figure 4.**
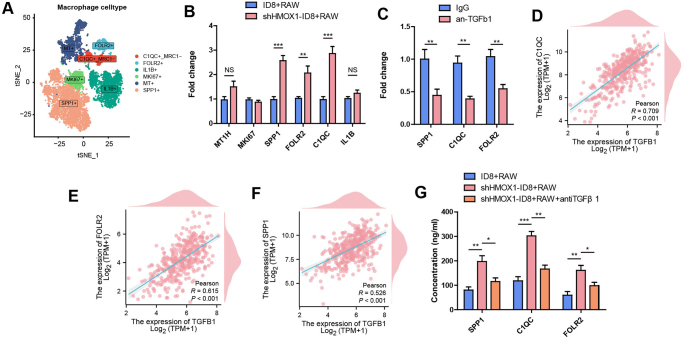
**TGF-β1 induces activation of SPP1^+^, FOLR2^+^, and C1QC^+^ subtype macrophages. (A)** t-SNE plot showing the six macrophage subtypes. Dots represent individual cells, and colors represent different cell populations. **(B)** mRNA of MT1H, SPP1, FOLR2, IL1B, C1QC and MKI67 were detected by RT-PCR in ID8 + RAW264.7 compared with shHMOX1-ID8 + RAW264.7. **(C)** mRNA of SPP1, FOLR, and C1QC were detected by RT-PCR in ID8 + RAW264.7 treated by IgG or anti-TGF-β1. **(D-F)** Correlation analysis of mRNA levels of TGF-β1 with SPP1, FOLR, and C1QC in TCGA-OV. **(G)** The concentration of SPP1, FOLR, and C1QC were detected by ELISA in ID8 + RAW264.7, shHMOX1-ID8 + RAW264.7 and shHMOX1-ID8 + RAW264.7 + anti-TGF-β1. *P < 0.05. **P < 0.01. ***P < 0.01.

Multiple macrophage subtypes were identified at the single-cell level. To investigate which subtypes were affected by TGF-β1 in the ovarian cancer tumor microenvironment, macrophage cells from GSE154600 were isolated and subjected to quality control following Seurat V5 standard procedures. These cells were classified into six distinct subtypes: C1QC^+^_MRC1^−^, MKI67^+^, FOLR2^+^, SPP1^+^, IL1B^+^, and MT^+^ macrophages (Fig. 4A). A co-culture model of ID8 and RAW264.7 was established, and the mRNA expression of C1QC, MKI67, SPP1, FOLR2, IL1B and MT1H (one key gene for each of the six subtypes) in RAW264.7 cells was detected by RT-PCR. The mRNA expression of C1QC, SPP1, and FOLR2 of RAW264.7 was higher in the sh-HMOX1 ID8 plus RAW264.7 group than in the ID8 plus RAW264.7 group (Fig. 4B). At the single-cell level, TISCH2 database showed that SPP1, FOLR2, and C1QC were mainly expressed on macrophages in eight datasets (Supplemental Digital Content Figure S3A, available at: http://links.lww.com/JS9/E551). As shown in Figure 4C, treatment with αTGF-β1 in co-culture conditions could reverse the mRNA expression of C1QC, SPP1, and FOLR2 of RAW264.7. Furthermore, a strong positive correlation in mRNA expression between C1QC, SPP1, and FOLR2 with TGF-β1 was observed in TCGA-OV (Fig. 4D-4F). Moreover, the concentrations of C1QC, SPP1, and FOLR2 were higher in the medium of the sh-HMOX1 ID8 plus RAW264.7 group than in the medium of the ID8 plus RAW264.7 group, and this increase could be abrogated by αTGF-β1 (Fig. 4G). During TIDE database analysis, among C1QC, SPP1, and FOLR2, only SPP1 showed a negative association with the activity of cytotoxic T lymphocytes or overall survival rates (Supplemental Digital Content Figures S3B and D, available at: http://links.lww.com/JS9/E551).

### TGF-β1 activates SPP1^+^, FOLR2^+^, and C1QC^+^ subtype macrophages via the PI3K/AKT/NF-κB(p65) axis to induce PD-1 inhibitor resistance

**Figure 5.**
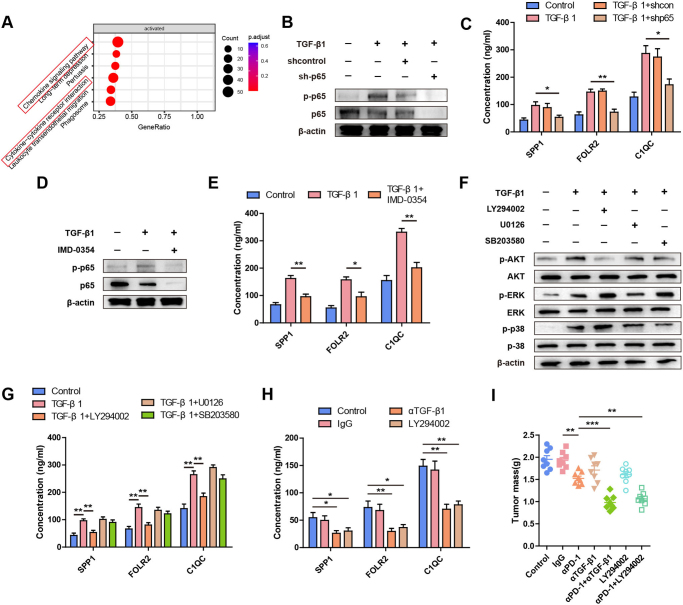
**TGF-β1 activates SPP1^+^, FOLR2^+^, and C1QC^+^ macrophage subtypes via the PI3K/AKT/NF-κB(p65) axis to induce PD-1 inhibitor resistance. (A)** Transcriptome analyses of RAW264.7 cells cultured with shHMOX1 ID8 cells or HMOX1-OE ID8 cells under non-contact-culture were conducted. All differential genes were assessed using Gene Set Enrichment Analysis (GSEA). **(B-C)** RAW264.7 cells were transfected with shcontrol or sh-p65 and exposed to TGF-β1 for 48 h. **(B)** Western blot analysis of p-p65, p65, and β-actin. **(C)** The concentrations of SPP1, FOLR, and C1QC in different groups were detected by ELISA. **(D-E)** RAW264.7 cells were treated with TGF-β1 or TGF-β1 + IMD-0354 for 48 h. **(D)** Western blot analysis of p-p65, p65, and β-actin. **(E)** The concentrations of SPP1, FOLR, and C1QC in different groups were detected by ELISA. **(F-G)** RAW264.7 were pretreated with inhibitors against PI3K (LY294002), ERK1/2 (U0126) or p38 (SB203580), followed by TGF-β1 treatment. **(F)** Expression levels of phosphorylated and total AKT, ERK and p38 were analyzed by western blot. **(G)** The concentrations of SPP1, FOLR, and C1QC in different groups were detected by ELISA. **(H)** The concentrations of SPP1, FOLR, and C1QC were detected by ELISA in control, IgG, αTGF-β1 or LY294002. **(I)** Tumor growth in ID8 tumor-bearing mice treated with saline, IgG, αPD-1, αTGF-β1, αPD-1 + αTGF-β1, LY294002 or αPD-1 + LY294002. N = 8 mice/group. Tumor growth was monitored by measuring the tumor volume for 2 weeks. *P < 0.05. **P < 0.01.

To investigate the mechanism by which TGF-β1 stimulates macrophages, we conducted transcriptome analyses of RAW264.7 cells cultured with shHMOX1 ID8 cells or HMOX1-OE ID8 cells under non-contact conditions (Only RAW264.7 cells from the two distinct co-cultures were compared). All differential genes were assessed using GSEA to identify candidate genes enriched in specific pathways. As depicted in Figure 5A, the chemokine signaling pathway and cytokine-cytokine receptor interaction were found to be involved, suggesting a potential link between cytokines and SPP1^+^, FOLR2^+^, and C1QC^+^ macrophage subtypes. NF-κB, a cytokine regulator known to be activated by TGF-β1, is a key molecule in tumor development and the maintenance of the M2 state in macrophages^[27]^. As illustrated in Figure 5B, exogenous TGF-β1 significantly upregulated the expression level of NF-κB (p-p65) in RAW264.7 cells. Knockout of NF-κB (p-p65) in macrophages effectively reversed TGF-β1-induced SPP1, FOLR2, and C1QC secretion (Fig. 5C). Similarly, the NF-κB pathway inhibitor (IMD-0354) significantly inhibited TGF-β1-induced SPP1, FOLR2, and C1QC secretion (Fig. 5D,E). To identify the signaling pathways involved in TGF-β1-induced secretion of SPP1, FOLR2, and C1QC by macrophages, we treated macrophages with inhibitors of PI3K, ERK, and p38 kinases, as these pathways can be activated by TGF-β1. Remarkably, PI3K inhibitors, but not ERK or p38 inhibitors, significantly suppressed TGF-β1-induced macrophage secretion of SPP1, FOLR2, and C1QC (Fig. 5F,G). *In vivo*, both TGF-β1 blocking antibody and LY294002 could decrease the concentration of SPP1, FOLR2, and C1QC (Fig. 5H). Collectively, these findings suggest that TGF-β1 activates SPP1^+^, FOLR2^+^, and C1QC^+^ macrophage subtypes through the PI3K/AKT/NF-κB (p-p65) pathway.

To investigate the impact of TGF-β1 or NF-κB (p-p65) on resistance to PD-1 inhibitors *in vivo*, we established a mouse model with tumor-bearing mice. The ID8 cells were inoculated and treated with various interventions, including IgG, PD-1 blocking antibody, TGF-β1 blocking antibody, PD-1 blocking antibody combined with TGF-β1 blocking antibody, LY294002, or PD-1 blocking antibody combined with LY294002. As depicted in Figure 5I, the tumor burden was significantly lower in the PD-1 blocking antibody plus TGF-β1 blocking antibody group or the PD-1 blocking antibody plus LY294002 group compared to the PD-1 blocking antibody group. Next, the concentrations of SPP1, FOLR2, and C1QC were detected in serum from the control, IgG, TGF-β1 blocking antibody and LY294002 groups.

### HMOX1^+^ macrophage induced PD-1 inhibitor resistance in ovarian cancer

**Figure 6.**
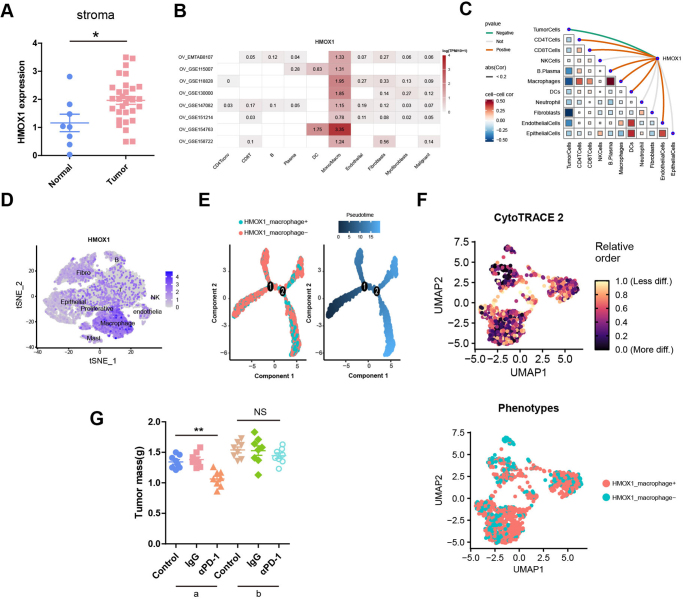
**HMOX1^+^ macrophage induced PD-1 inhibitor resistance in ovarian cancer. (A)** The expression of HMOX1 in tumor stroma compared with normal stroma in GSE40595. **(B)** Analysis of HMOX1 expression in different cells in ovarian cancer by the TISCH2 database. **(C)** Spearman correlation between HMOX1 expression and microenvironmental components in GSE203612. **(D)** t-SNE plot showing expression of HMOX1 in GSE154600. **(E)** Monocle plots showing the differentiation trajectories of HMOX1_macrophage^+^ and HMOX1_ macrophage^−^ cells. **(F)** Combined application of CytoTRACE to dissect HMOX1_macrophage^+^ and HMOX1_ macrophage^−^ cells. **(G)** Tumor growth in mice bearing a or b, Mice were given saline, IgG or αPD-1. N = 8 mice/group. Tumor growth was monitored by measuring the tumor volume for 2 weeks. (a = ID8 plus RAW264.7 group, b = ID8 plus HMOX1-OE RAW264.7 group). *P < 0.05. **P < 0.01.

To determine whether HMOX1 is also expressed in other cell types, we further analyzed dataset GSE40595. We found that the mRNA expression of HMOX1 in tumor stroma was higher than on normal stroma (Fig. 6A). At the single-cell level, TISCH2 database analysis revealed that HMOX1 was mainly expressed in macrophages in eight datasets (Fig. 6B). In the spatial transcriptome dataset (GSE203612), the expression of HMOX1 was negatively correlated with tumor cells and positively correlated with macrophages, CD4T, CD8T and B cells (Fig. 6C). Transcriptome, single-cell and spatial transcriptome datasets confirmed that only macrophages in the stroma exhibited high expression of HMOX1. Spatial transcriptome data showed that HMOX1 was positively correlated with CD4T, CD8T and B cells. However, in single-cell data, neither T nor B cells expressed HMOX1, confirming that high expression of HMOX1 in other cells activates CD4 + T, CD8 + T, and B cells, consistent with our previous experimental results.

Next, we further analyzed the function of HMOX1 on macrophages. As shown in Figure 6D, HMOX1 was also mainly expressed by macrophages in GSE154600. To gain insights into the dynamic processes underlying HMOX1 expression on macrophages at the single-cell level, macrophage cells were subset from the total cell population. Macrophages expressing HMOX1 were designated HMOX1_macrophage^+^, while those without HMOX1 were termed HMOX1_macrophage^−^. The pseudotime cell trajectory of HMOX1_macrophage^+^ and HMOX1_macrophage^−^ was derived based on the Monocle 2 algorithm. HMOX1_macrophage^−^ was present mainly in the progenitor state, while HMOX1_macrophage^+^ was present in the terminal state (Fig. 6E). Next, CytoTRACE was applied to delineate cellular hierarchies based on HMOX1_macrophage^+^ and HMOX1_macrophage^−^. The results indicated HMOX1_macrophage^+^ were predominantly in a more differentiated state (Fig. 6F). These results collectively indicate that as ovarian cancer progresses, the proportion of macrophages with high HMOX1 expression gradually increases.

To investigate the impact of HMOX1 on macrophage-mediated responses to PD-1 inhibitors, we established a tumor-bearing mouse model. Mice were inoculated with either ID8 plus RAW264.7 cells or ID8 plus HMOX1-OE RAW264.7 cells and treated with IgG or a PD-1 blocking antibody. As depicted in Figure 6G, anti-PD-1 antibody treatment effectively inhibited tumor growth in the ID8 plus RAW264.7 cells model compared to IgG. However, no significant difference was observed between IgG and anti-PD-1 antibody treatment in the ID8 plus HMOX1-OE RAW264.7 model, indicating that macrophages with high expression of HMOX1 induced PD-1 inhibitor resistance.

### HMOX1^+^ macrophages in ovarian cancer promote the SPP1^+^, FOLR2^+^, and C1QC^+^ subtypes

**Figure 7.**
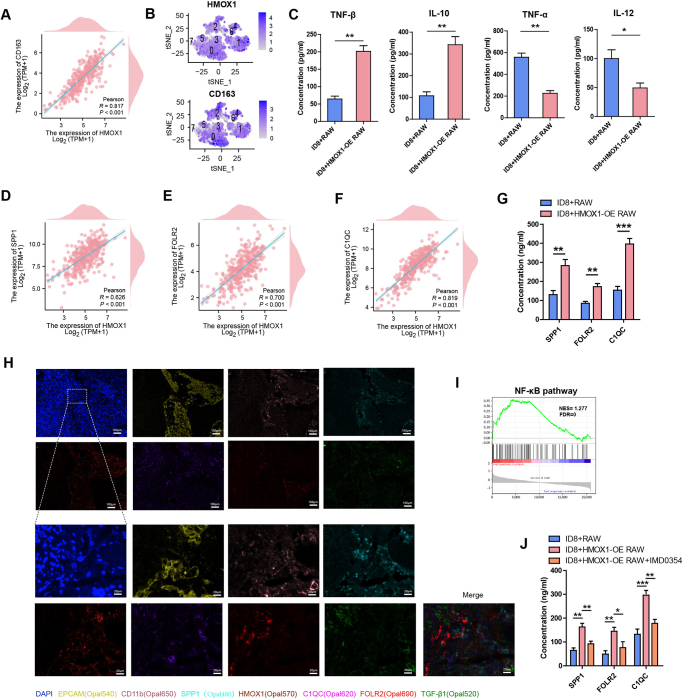
**HMOX1^+^ macrophage in ovarian cancer activate SPP1^+^, FOLR2^+^, and C1QC^+^ subtypes. (A)** Correlation analysis of mRNA levels of HMOX1 with CD163 in TCGA-OV. **(B)** t-SNE plot showing expression of HMOX1 and CD163 on macrophages in GSE154600. **(C)** The concentrations of TNF-α, IL-10, IL-12, and TNF-β in the supernatant of ID8 coculture with HMOX1-OE-RAW264.7 or ID8 coculture with RAW264.7 were detected by ELISA. **(D-F)** Correlation analysis of mRNA levels of HMOX1 with SPP1, FOLR2, and C1QC in TCGA-OV. **(G)** The concentrations of SPP1, FOLR2, and C1QC in the supernatant of ID8 coculture with HMOX1-OE-RAW264.7 or ID8 coculture with RAW264.7 were detected by ELISA. **(H)** Multi-color fluorescence co-localization detection of HMOX1, TGF-β1, SPP1, FOLR2, and C1QC. **(I)** GSEA analysis of the NF-κB pathway in cancer stroma in GSE40595 (All cancer stroma specimens were divided into high and low groups according to the mean expression of HMOX1). **(J)** The concentrations of SPP1, FOLR2, and C1QC in the supernatant of ID8 coculture with HMOX1-OE-RAW264.7, ID8 coculture with RAW264.7 or ID8 coculture with HMOX1-OE-RAW264.7 treated by IMD0354 were detected by ELISA.

A strong positive correlation between HMOX1 and CD163 mRNA expression was observed in TCGA ovarian cancer clinical specimens (Fig. 7A). In dataset GSE154600, significant overlap was observed between HMOX1 and CD163 (Fig. 7B). Furthermore, to investigate the effect of HMOX1 in macrophage polarization, ELISA was conducted to detect IL-10, TNF-α, IL-12, and TNF-β expression. Notably, the concentration of IL-10 and TNF-β from the ID8 plus HMOX1-OE-RAW264.7 group was significantly higher than that from the ID8 plus RAW264.7 group, while the results for TNF-α and IL-12 displayed an inverse pattern (Fig. 7C). These outcomes indicate that heightened HMOX1 expression sustains the M2 macrophage phenotype.

**Figure 8.**
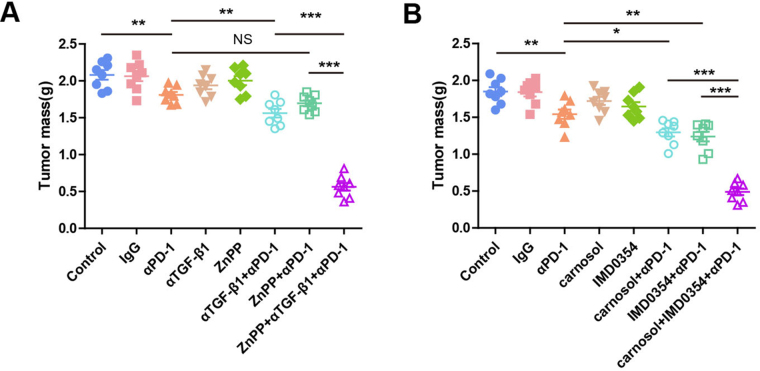
**Targeting HMOX1 improves the efficacy of PD-1 inhibitors. (A)** Tumor growth in ID8 tumor-bearing mice treated with saline, IgG, αPD-1, αTGF-β1, ZnPP, αTGF-β1 + αPD-1, ZnPP + αPD-1 or ZnPP + αTGF-β1 + αPD-1. N = 8 mice/group. Tumor growth was monitored by measuring the tumor volume for 2 weeks. **(B)** Tumor growth in mice bearing ID8, Mice were given saline, IgG, αPD-1, carnosol, IMD0354, carnosol + αPD-1, IMD0354 + αPD-1 or carnosol + IMD0354 + αPD-1. N = 8 mice/group. Tumor growth was monitored by measuring the tumor volume for 2 weeks.

We next investigated whether HMOX1 regulates the expression of macrophage-associated genes *SPP1, FOLR2*, and *C1QC*. Transcriptional analysis of TCGA-OV specimens showed significant co-expression patterns (Fig. 7D-7F), which were validated at the protein level in our HMOX1-overexpression model (Fig. 7G). Multicolor fluorescence co-localization microscopy of ovarian cancer specimens revealed the distinct cellular location of specific markers. To precisely identify epithelial cells and macrophages, EPCAM and CD11b markers were utilized, respectively. The results also confirmed that HMOX1, SPP1, FOLR2, and C1QC were mainly expressed in macrophages with a high degree of overlap, while TGF-β1 was expressed at the periphery of these co-localization sites (Fig. 7H).

As shown in Figure 5, TGF-β1 could activate SPP1^+^, FOLR2^+^, and C1QC^+^ macrophage subtypes via the NF-κB signaling pathway. Accordingly, we sought to investigate whether HMOX1 utilizes a similar mechanism to activate these three macrophage subtypes. Utilizing GSEA, we assessed the NF-κB signaling pathway in dataset GSE40595. All tumor stroma samples were divided into low HMOX1 and high HMOX1 groups based on the mean mRNA expression of HMOX1. As shown in Figure 7I, comparison between the two groups revealed positive regulation of the NF-κB signaling pathway in ovarian stroma (NES = 1.277, FDR = 0). Besides, the NF-κB signaling pathway inhibitor (IMD0354) could decrease the concentration of SPP1, FOLR2, and C1QC in ID8 + HMOX1-OE RAW group. These data suggested that HMOX1 exerts opposing effects on macrophages compared to epithelial cells (Fig. 7J).

### Targeting HMOX1 improves the efficacy of PD-1 inhibitors

To assess the impact of HMOX1 on D-1 inhibitor efficacy, we established two tumor-bearing mouse models. Given HMOX1’s opposite effects in macrophages and epithelial cells, we employed dual strategies: (1) ZnPP was used to down-regulate the expression of HMOX1 in the whole tumor, and TGF-β1 blocking antibody was used to block the secretion of TGF-β1 activated by inhibition of HMOX1 in epithelium; (2) carnosol was used to up-regulate HMOX1 expression in the whole tumor, and IMD0354 was used to block the NF-κB pathway activated by HMOX1 up-regulation in macrophages. In the first model, ID8 cell-bearing mice received IgG, PD-1 blockade, TGF-β1 blockade, ZnPP, or their combinations. As depicted in Figure 8A, ZnPP + PD-1 blockade alone failed to reduce tumor burden versus PD-1 blockade, whereas ZnPP + TGF-β1 + PD-1 blockade significantly outperformed either dual therapy. In the second model, the ID8 cells were inoculated and treated with IgG, PD-1 blocking antibody, IMD0354, carnosol, IMD0354 + PD-1 blocking antibody, carnosol + PD-1 blocking antibody or carnosol + IMD0354 blocking antibody + PD-1 blocking antibody. As depicted in Figure 8B, tumor burden was significantly decreased with carnosol + IMD0354 + PD-1 blockade compared to IMD0354 + PD-1 blockade or carnosol + PD-1 blockade. These results confirmed HMOX1’s cell type-dependent antagonistic roles *in vivo* and revealed that isolated HMOX1 modulation induces a dual-edged effect. Optimal therapeutic outcomes thus require combinatorial strategies to mitigate compensatory pathways.

### HTSFC can predict the efficacy of PD-1 inhibitors in ovarian cancer

**Figure 9.**
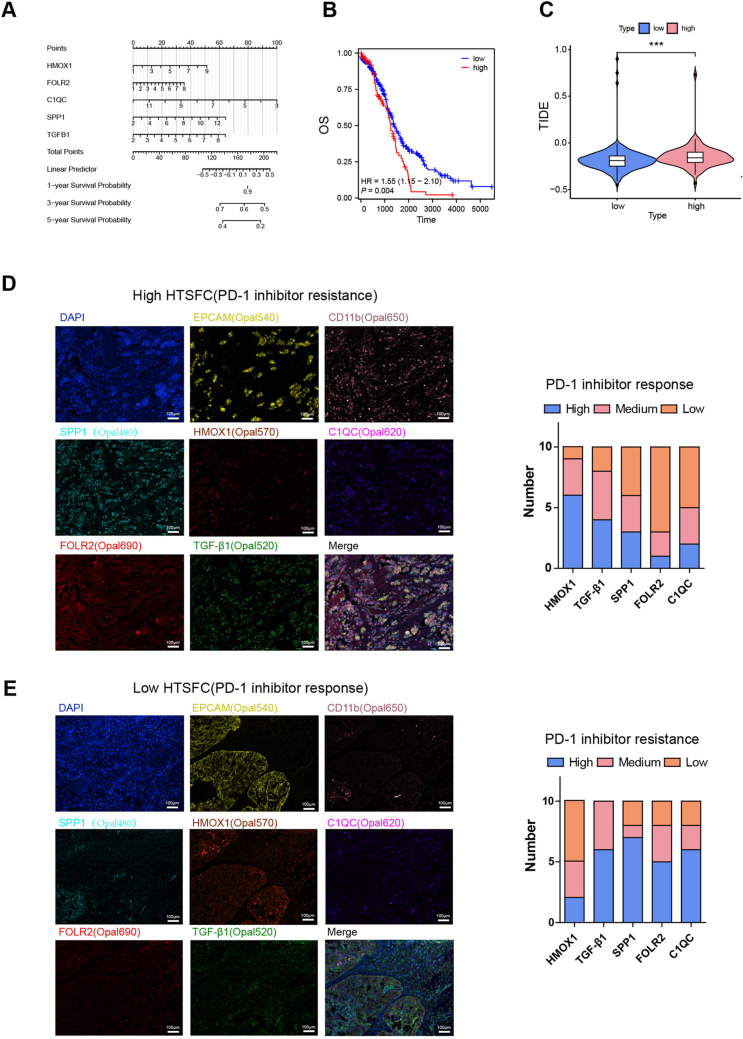
**HTSFC can predict the efficacy of PD-1 inhibitors in ovarian cancer. (A)** A nomogram was established to predict the prognosis of TCGA-OV patients. **(B)** Overall survival of TCGA-OV patients stratified in high and low-risk groups using the nomogram. **(C)** TIDE analysis for high and low-risk groups. **(D)** The fluorescence intensity of HMOX1, TGF-β1, SPP1, FOLR2, and C1QC were calculated in PD-1 inhibitor response. **(E)** The fluorescence intensity of HMOX1, TGF-β1, SPP1, FOLR2, and C1QC were calculated in PD-1 inhibitor resistance clinical specimens. ***P < 0.01.

To evaluate the effects of HMOX1, TGF-β1, SPP1, FOLR2, and C1QC on ovarian cancer survival and the efficacy of PD-1 inhibitors, we constructed a prognostic nomogram model in the TCGA cohort to predict the 1-, 3-, and 5-year overall survival (OS) of OV for predicting the 1-, 3-, and 5-year survival of OV patients (Fig. 9A). According to the risk score, TCGA-OV specimens were stratified into 2 groups: the highest 25% was classified as the high-risk group, and the remaining constituted the low-risk group. The high-risk group exhibited a significant negative correlation with overall survival (Fig. 9B). Next, the TIDE algorithm was used to predict the efficacy of checkpoint inhibitors in TCGA-OV. The results showed that the high-risk group yielded significantly higher scores than the low-risk group, indicating tolerance to checkpoint inhibitors in the high-risk group (Fig. 9C). These results suggest that the combined expression of HMOX1, TGF-β1, SPP1, FOLR2, and C1QC serves as a robust predictor of ovarian cancer survival and immune checkpoint resistance. Accordingly, this five-gene signature was designated as “HTSFC.”

Twenty clinical specimens collected before receiving PD-1 inhibitor treatment were used to evaluate the expression of HTSFC, with detailed information shown in Table 2. Multiplex immunofluorescence staining was performed to evaluate DAPI, EPCAM, CD11B, HMOX1, TGF-β1, SPP1, FOLR2, and C1QC expression in each specimen. As shown in Figure 9D, TGF-β1, SPP1, FOLR2, and C1QC exhibited intermediate-to-high expression in the samples of PD-1 inhibitor resistance patients, while the low-to-intermediate expression was observed in the samples of PD-1 inhibitor response patients, which confirmed HTSFC’s excellent ability to predict response to PD-1 inhibitor in ovarian cancer (Fig. 9E).

**Table 2:** 

*sample_name	*organism	*isolate	*age	*biomaterial_provider	*collection_date	*tissue
Immunology resist-1	Homo sapiens	Ovarian	54	Department of Oncology, XiangYang Central Hospital	2020.7	Ovarian adenocarcinoma
Immunology resist-2	Homo sapiens	Ovarian	46	Department of Oncology, XiangYang Central Hospital	2020.1	Ovarian adenocarcinoma
Immunology resist-3	Homo sapiens	Peritoneal	61	Department of Oncology, XiangYang Central Hospital	2020.3	Ovarian adenocarcinoma
Immunology resist-4	Homo sapiens	Ovarian	57	Department of Oncology, XiangYang Central Hospital	2020.4	Ovarian adenocarcinoma
Immunology resist-5	Homo sapiens	Ascites	49	Department of Oncology, XiangYang Central Hospital	2020.6	Ovarian adenocarcinoma
Immunology resist-6	Homo sapiens	Ovarian	52	Department of Oncology, XiangYang Central Hospital	2020.6	Ovarian adenocarcinoma
Immunology resist-7	Homo sapiens	Peritoneal	68	Department of Oncology, XiangYang Central Hospital	2019.12	Ovarian adenocarcinoma
Immunology resist-8	Homo sapiens	Peritoneal	62	Department of Oncology, XiangYang Central Hospital	2019.1	Ovarian adenocarcinoma
Immunology resist-9	Homo sapiens	Ovarian	70	Department of Oncology, XiangYang Central Hospital	2019.7	Ovarian adenocarcinoma
Immunology resist-10	Homo sapiens	Ascites	49	Department of Oncology, XiangYang Central Hospital	2020.3	Ovarian adenocarcinoma
Immunology activity-1	Homo sapiens	Ascites	58	Department of Oncology, XiangYang Central Hospital	2019.1	Ovarian adenocarcinoma
Immunology activity-2	Homo sapiens	Ascites	72	Department of Oncology, XiangYang Central Hospital	2019.8	Ovarian adenocarcinoma
Immunology activity-3	Homo sapiens	Peritoneal	65	Department of Oncology, XiangYang Central Hospital	2019.7	Ovarian adenocarcinoma
Immunology activity-4	Homo sapiens	Peritoneal	63	Department of Oncology, XiangYang Central Hospital	2019.7	Ovarian adenocarcinoma
Immunology activity-5	Homo sapiens	Peritoneal	52	Department of Oncology, XiangYang Central Hospital	2020.3	Ovarian adenocarcinoma
Immunology activity-6	Homo sapiens	Ovarian	64	Department of Oncology, XiangYang Central Hospital	2020.5	Ovarian adenocarcinoma
Immunology activity-7	Homo sapiens	Ovarian	57	Department of Oncology, XiangYang Central Hospital	2021.3	Ovarian adenocarcinoma
Immunology activity-8	Homo sapiens	Peritoneal	48	Department of Oncology, XiangYang Central Hospital	2020.12	Ovarian adenocarcinoma
Immunology activity-9	Homo sapiens	Ovarian	55	Department of Oncology, XiangYang Central Hospital	2019.7	Ovarian adenocarcinoma
Immunology activity-10	Homo sapiens	Ovarian	62	Department of Oncology, XiangYang Central Hospital	2019.1	Ovarian adenocarcinoma

The detailed inf-ormation of samples from twenty ovarian patients for the evaluation the expression of HTSFC.

## Discussion

While immunotherapy has achieved success in certain malignancies, its efficacy in ovarian cancer remains limited, primarily attributed to the presence of a complex immunosuppressive tumor microenvironment^[28–30]^. Ferroptosis, an iron-dependent form of cell death triggered by lipid peroxidation and excessive ROS production, has been linked to tumor progression and therapeutic response across various tumors^[31,32]^. The precise role of ferroptosis, however, remains contentious, with conflicting findings reported not only across different cancer types but also within the same malignancy, leading to disparate conclusions from various research teams^[14,16,33]^. In this study, we employed transcriptome sequencing, single-cell spatial transcriptomics, and clinical specimens treated with PD-1 inhibitors, along with *in vivo* and *in vitro* experiments, to uncover the “double-edged sword” mechanism of the ferroptosis-activated gene *HMOX1* in the immune microenvironment.

In this study, the PD-1 inhibitor-resistant cell line ID8R was obtained through animal models. Transcriptome sequencing identified eight differentially expressed genes that intersected with ferroptosis-related genes. A comparison of the expression of two recognized ferroptosis suppressor genes (*GPX4* and *SLC7A11*) in the TCGA-OV and GTEx databases indicated that the ferroptosis pathway in ovarian cancer was inhibited, consistent with previous reports^[34,35]^. Further validation involved comparing ROS production in ID8 and ID8R cells under the influence of ferroptosis inhibitors and activators. This confirmed that ID8R cells exhibited a state of ferroptosis inhibition relative to ID8. Therefore, mRNA expression levels of ferroptosis-associated genes (HMOX1 and NOX1) were significantly downregulated in ID8R cells, suggesting their potential role as key regulatory genes in this process. WB results showed that only HMOX1 protein levels were significantly inhibited in ID8R. HMOX1, an inducible intracellular enzyme responsible for degrading heme and releasing biliverdin, carbon monoxide (CO) and free ferrous iron, is considered a key gene in ferroptosis^[36,37]^. HMOX1 has been shown to be involved in various immune states and associated with the efficacy of immune checkpoint inhibitors^[38–40]^. Analyses using KM-plot and TIDE database, in conjunction with our own clinical specimens treated with PD-1 inhibitors, confirmed that downregulation of HMOX1 expression in ovarian cancer may be a key gene associated with PD-1 inhibitor resistance.

However, a previous study suggested that in PTEN-deficient ovarian cancers, HMOX1-expressing macrophages significantly promote tumor progression^[18]^. This finding, however, does not align with our results. Compared with TCGA-OV and GTEx, the mRNA expression of HMOX1 in ovarian cancer tissues was significantly lower than that in normal ovarian tissues. In GSE40595, mRNA levels of HMOX1 were significantly lower in tumor epithelium than in normal epithelium, and significantly higher in tumor stroma than in normal stroma. Spatial transcriptome data showed that HMOX1 expression was lower at the tumor site than at the junction site, and lower at the junction site than at the normal site, which indicated that the down-regulation of HMOX1 expression in ovarian cancer mainly originated from epithelial cells. TISCH database analysis showed that HMOX1 was mainly expressed in macrophages. Analysis of the single-cell dataset GSE154600 revealed that HMOX1 was also mainly expressed in macrophages. Pseudo-time series analysis of these two cell populations showed that in the process of ovarian cancer progression, the tumor epithelium was predominantly composed of HMOX1-malignant^−^ cells, while HMOX1-macrophage^+^ cells accounted for the vast majority of macrophages, which confirmed that both HMOX1-malignant^−^ cells and HMOX1-macrophage^+^ cells become dominant cells in the late stage of ovarian cancer progression. These results suggested that in the progression of ovarian cancer, HMOX1 may perform different functions in tumor epithelium and macrophages.

Immune checkpoint inhibitors function by activating CTLs for tumor elimination^[41]^. In ovarian cancer, characterized by an immunosuppressive microenvironment, immune checkpoint inhibitors struggle to effectively activate CTLs, resulting in poor clinical outcomes^[30,42,43]^. In our clinical samples, cytotoxic CD8^+^T cells were significantly decreased in the low HMOX1 expression group compared to the high HMOX1 expression group. In a tumor-bearing mouse model, the shHMOX1 group showed significant decreases in CD4^+^/CD8^+^ ratios and tumor-specific T cells. These results demonstrated that inhibition of HMOX1 in ovarian epithelium reduced CTL activation. Cytokine protein microarray results showed that ovarian cancer epithelium with low HMOX1 expression exhibited significantly increased secretion of TGF-β1. Previous studies have shown that TGF-β1 is involved in the immune escape of tumor cells, and TGF-β1 is significantly upregulated in clinical specimens resistant to PD-1/PD-L1 inhibitors^[44,45]^. TGF-β1 has been shown to regulate myeloid cells and immune cells, especially M2-type macrophages, in the tumor immune microenvironment^[26,46,47]^. Herein, we demonstrated that ovarian cancer epithelial cells with low expression of HMOX1 promoted macrophage M2 polarization. Unlike prior approaches that treated macrophages as a monolithic research pathway, our investigation underscored the heterogeneity inherent in macrophage populations, given that multiple subtypes have been empirically demonstrated to critically influence both tumor progression and immunotherapeutic efficacy. Leveraging dataset GSE154600, we obtained six macrophage subtypes (SPP1^+^, FOLR2^+^, C1QC^+^, MKI67^+^, MT^+^ and IL1B^+^). PCR and ELISA confirmed that ovarian cancer epithelial cells with low HMOX1 expression mainly affected three subgroups of macrophages (SPP1^+^, FOLR2^+^, and C1QC^+^), and blocking TGF-β1 antibodies reversed this phenomenon. SPP1, FOLR2, and C1QC were also expressed almost exclusively on macrophages and have been shown to be key subgroups of tumor-associated macrophages (TAM) in tumors^[48,49]^. In TCGA-OV, TGF-β1 was highly correlated with SPP1, FOLR2, and C1QC. The above findings suggest that the inhibition of HMOX1 in ovarian cancer epithelial cells can lead to significant secretion of TGF-β1, thereby affecting the SPP1^+^, FOLR2^+^, and C1QC^+^ macrophage subsets.

Exploring the mechanism by which low HMOX1 expression in ovarian cancer epithelial cells regulates these three macrophage subtypes can provide a more precise target for improving the efficacy of PD-1 inhibitors. KEGG analysis showed that cytokine signaling pathways and cytokine receptor pathways were significantly activated in macrophages stimulated by TGF-β1. NF-κB, a cytokine regulator that fosters M2-type macrophage polarization in tumors, plays a role in tumor immune evasion^[50,51]^. An interaction between the TGF-β1 and NF-κB pathways was observed, highlighting their mutual regulation^[52]^. NF-κB can be activated through three pathways – MAPK, ERK, and ATK^[53]^. Subsequent experiments confirmed that TGF-β1 induced macrophage-secreted SPP1, FOLR2, and C1QC by activating the PI3K/AKT/NF-κB(p-p65) axis. Animal models further validated that blocking TGF-β1 or AKT could decrease the concentrations of SPP1, FOLR2, and C1QC, and enhance the efficacy of PD-1 inhibitors.

Next, we sought to elucidate the role of elevated HMOX1 expression by macrophages during ovarian cancer progression and its capacity to modulate the immune microenvironment. Initial analyses, encompassing both bulk and single-cell sequencing data, corroborated a significant correlation between HMOX1 and CD163. Moreover, macrophages characterized by high HMOX1 expression presented with an M2-like morphology. Prompted by the observation that increased HMOX1 expression in macrophages influences their polarization, we sought to determine its effect on specific macrophage subtypes. Our findings substantiated a robust correlation between HMOX1 and SPP1, FOLR2, and C1QC, contrasting with only a marginal correlation observed with IL1B, MT1H, and MKI67 (data not presented). Since this study confirmed that the NF-κB pathway is key to upregulating these three subgroups, we performed GSEA to examine NF-κB pathway enrichment in dataset GSE40595 samples categorized by high and low HMOX1 expression. The results showed that this pathway was positively correlated in the group with high HMOX1 expression. At the same time, NF-κB inhibitors could block the concentration of SPP1, FOLR2, and C1QC from macrophages with high expression of HMOX1. Since HMOX1 is expressed almost exclusively on macrophages, the above results confirmed that high expression of HMOX1 in macrophages upregulates the differentiation of NF-κB in the SPP1, FOLR2, and C1QC subtypes. To date, our investigations have established that in ovarian cancer, HMOX1 exhibits contrasting expression patterns between epithelial cells and macrophages, yet its functional contribution to fostering an immunosuppressive microenvironment remains consistent. This finding not only provides a coherent resolution to the apparent discrepancies in prior ferroptosis-related research but also suggests that this phenomenon may have broader applicability. Consequently, therapeutic strategies focused on modulating a single gene, whether through suppression or activation alone, may inadvertently trigger compensatory feedback mechanisms, resulting in a “double-edged effect.”

Overall, this study utilized multicenter and multi-omics data to confirm that the ferroptosis-activating gene HMOX1 exhibits two opposite expression states in ovarian cancer epithelial cells and macrophages, and synergistically induces the differentiation of three macrophage subtypes through the NF-κB pathway, thereby contributing to PD-1 inhibitor resistance. Current evidence highlights the necessity for clinical research and translational efforts to emphasize investigating gene- and pathway-specific effects across distinct cell types. There is compelling rationale to hypothesize that this biological phenomenon, though potentially widespread in clinical settings, has historically been overlooked. Accordingly, the findings of this study may warrant validation across a broader spectrum of disease contexts. The implementation of the HTSFC-based model represents a methodological advancement that significantly strengthens this work, thereby expanding its potential clinical utility. Such applications may comprise, but are not limited to, diagnostic kits targeting the five identified genes through PCR-based assays, immunohistochemical techniques, or rapid colloidal gold immunochromatography platforms.

However, this study has several limitations that should be acknowledged. First, despite leveraging autonomous transcriptome sequencing, multiple transcriptomes from different centers, single-cell sequencing, and spatial transcriptomes to support its conclusions, the number of specimens was relatively small. Besides, this study focused on ovarian cancer as a whole and did not consider its different stages and types. Moreover, only 20 clinical specimens were used to verify HTSFC, and no prospective studies were conducted. Consequently, its ultimate clinical translational capability requires further verification.

## Data Availability

Data are available upon request. Most results from this study are included in the article.
